# Cancer care delivery during the Paris 2024 Olympic and Paralympic games: lessons from a crisis-driven Hospital at Home program

**DOI:** 10.3389/fmed.2025.1560027

**Published:** 2025-03-31

**Authors:** Jeremie Zerbit, Cecile Chauvin, Arsene Zogo, David Avran, Frederique Moufle, Florent Bousquie, Clement Leclaire

**Affiliations:** Greater Paris University Hospitals (AP-HP), Paris Public Hospital at Home (HAD AP-HP), Paris, France

**Keywords:** Hospital at Home, cancer care, Olympic games, healthcare logistics, continuity of care, oncology treatment, crisis management, home-based chemotherapy

## Abstract

**Introduction:**

The Paris 2024 Olympic and Paralympic Games posed logistical challenges for healthcare delivery, particularly for maintaining home-based cancer treatments amidst road closures and 15 million visitors. The Hospital at Home (HaH) program of Greater Paris University Hospitals (AP-HP) implemented innovative strategies to ensure uninterrupted care during this period.

**Methods:**

HaH deployed a “Games Pass” system for its fleet of 500 vehicles and introduced electric bicycles and optimized pedestrian routes to address accessibility constraints. Dedicated lanes, in collaboration with city officials, ensured timely care. Personalized care plans were developed, accounting for patient locations and event schedules. Drug preparation was centralized, and advanced cold-chain methods facilitated delivery. Strategic pre-positioning of vehicles and personnel within restricted zones ensured continuous care, supported by real-time coordination through a dedicated management team.

**Results:**

Between July 26 and September 8, 2024, HaH administered 1,946 chemotherapy/immunotherapy sessions to 535 patients (median age 72, IQR 60–79), including 29 pediatric cases. This represented 4.76% of AP-HP’s total treated cancer patients, a significant increase from 2023 (3.9%, *p* < 0.05). Treatments included 31 drugs, with azacitidine (*n* = 1,025) and daratumumab (*n* = 248) being most common. Key indications were multiple myeloma (*n* = 235) and myeloid neoplasms (*n* = 175). No treatment delays or patient harm were reported.

**Conclusion:**

The HaH program ensured continuity of care during the Games, highlighting the importance of flexibility, real-time problem-solving, and patient-centered planning. These strategies offer valuable insights for improving routine HaH operations and managing healthcare during large-scale events.

## Introduction

The Paris 2024 Olympic and Paralympic Games (OG & PG) posed significant challenges to the healthcare infrastructure, especially in maintaining critical treatments like home-based cancer care. With 15 million visitors and extensive road closures expected, AP-HP coordinated closely with state services and the Paris 2024 Organizing Committee. The Hospital at Home (HaH) of Greater Paris University Hospitals (AP-HP) responded with strategic measures, drawing on past crisis experiences such as the COVID-19 pandemic (1, 2). These events underscored the critical role of preemptive planning and adaptive logistics in ensuring continuity of care, reinforcing HaH’s capacity to provide home-based treatments under challenging conditions (3, 4). Leveraging these insights, personalized care plans were developed to optimize treatment delivery while navigating the unique logistical constraints of the Games.

## Methods

AP-HP is the largest public hospital network in Europe, encompassing 38 hospitals, including a HaH program. AP-HP treats approximately 8 million patients annually, with its HaH program managing around 15,000 stays per year for the administration of injectable anticancer treatments.

### Anticipating accessibility challenges

A “Games Pass” system was introduced for the fleet of 500 vehicles, ensuring access to restricted areas. The use of electric bicycles and optimized pedestrian routes further mitigated traffic constraints. Collaborations with city officials and law enforcement allowed dedicated lanes for HaH vehicles, ensuring timely care delivery.

### Tailored care and optimized schedules

Personalized care plans were developed for each patient, considering their location and the timing of Olympic events. On critical days, such as the Opening Ceremony and major sports events, the preparation and delivery of injectable anticancer drugs were meticulously planned, with centralized drug preparation in a single hospital near major restricted zones and advanced delivery using specialized cold-chain methods. Home-based clinical evaluations for medical clearance were conducted before treatment days.

### Cold-chain methods

For the secure transport of temperature-sensitive anticancer drugs, we employed validated cold-chain solutions, including passive insulated containers with phase-change materials ensuring stable temperatures for up to 72 h. Additionally, real-time temperature monitoring was integrated to prevent deviations during transit.

### Strategic pre-positioned resources

Vehicles and healthcare personnel were strategically placed within affected zones to ensure timely treatment delivery. These vehicles acted as mobile logistical units and on-site reserves, enabling continued patient care within closed loops for several hours, even in otherwise inaccessible areas. Each healthcare provider was assigned a specific sector and role, mirroring the teamwork seen in Olympic teams.

### Patient and public involvement

Patients were involved in the design of the study by providing input on the research objectives and protocol feasibility. Additionally, patients will be engaged in the dissemination of results through presentations at patient advocacy groups.

### Institutional coordination

The development and implementation of this program were conducted in collaboration with the AP-HP headquarters and its strategic departments, the regional health agency (ARS Ile-de-France), the Paris 2024 Organizing Committee, the Prefecture of Police, and the City of Paris.

## Results

**Figure 1:**
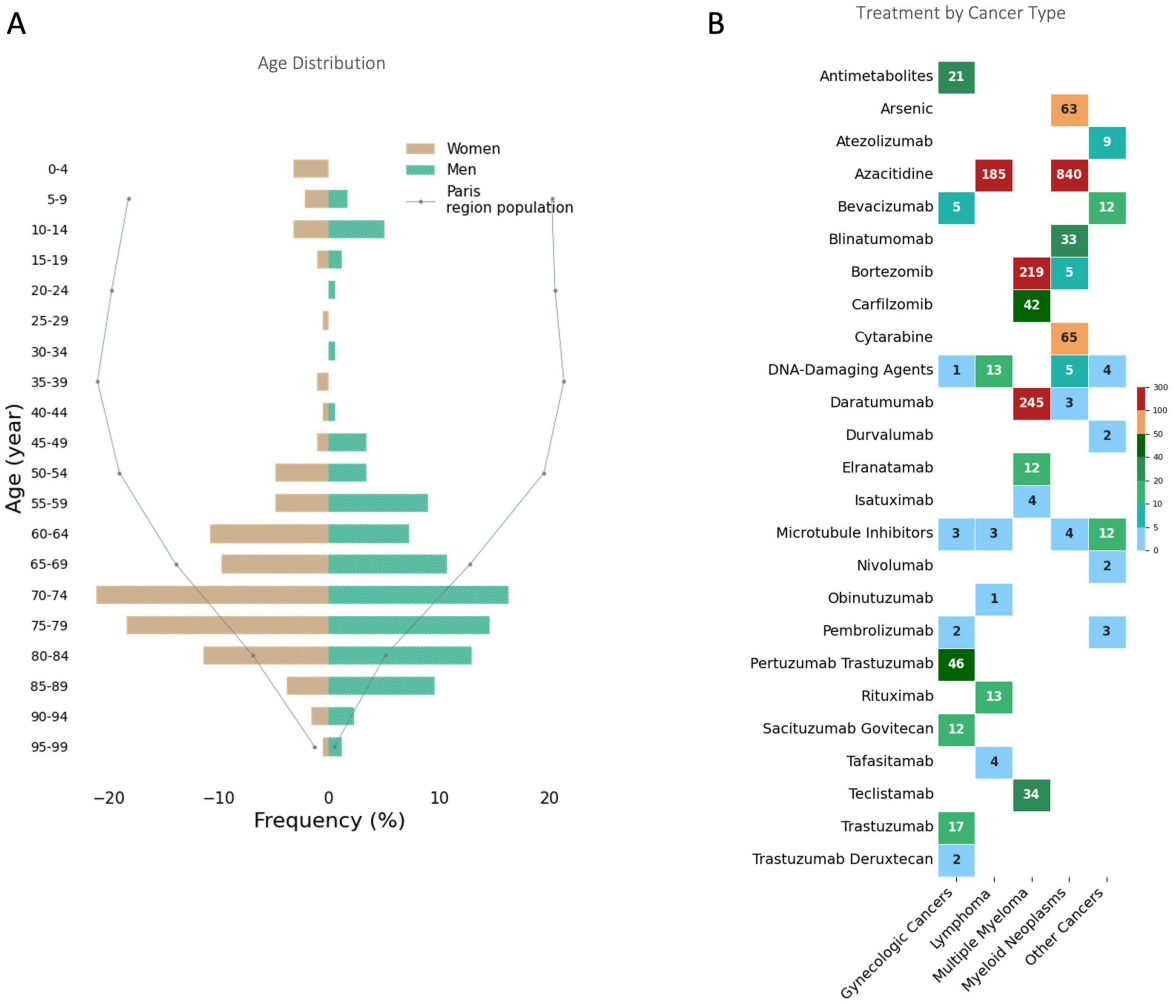
Age Distribution and Treatment by Cancer Type in the Paris Region During the 2024 Olympics. **(A)** Age distribution of patients receiving Hospital at Home cancer treatments during the 2024 Paris Olympics. The Paris region population distribution is shown as a reference (dotted line). **(B)** Number of treatments administered for each cancer type, categorized by therapeutic agents. The data are grouped into major cancer categories. The color gradient indicates the frequency of treatments, with darker colors representing higher values.

**Figure 2:**
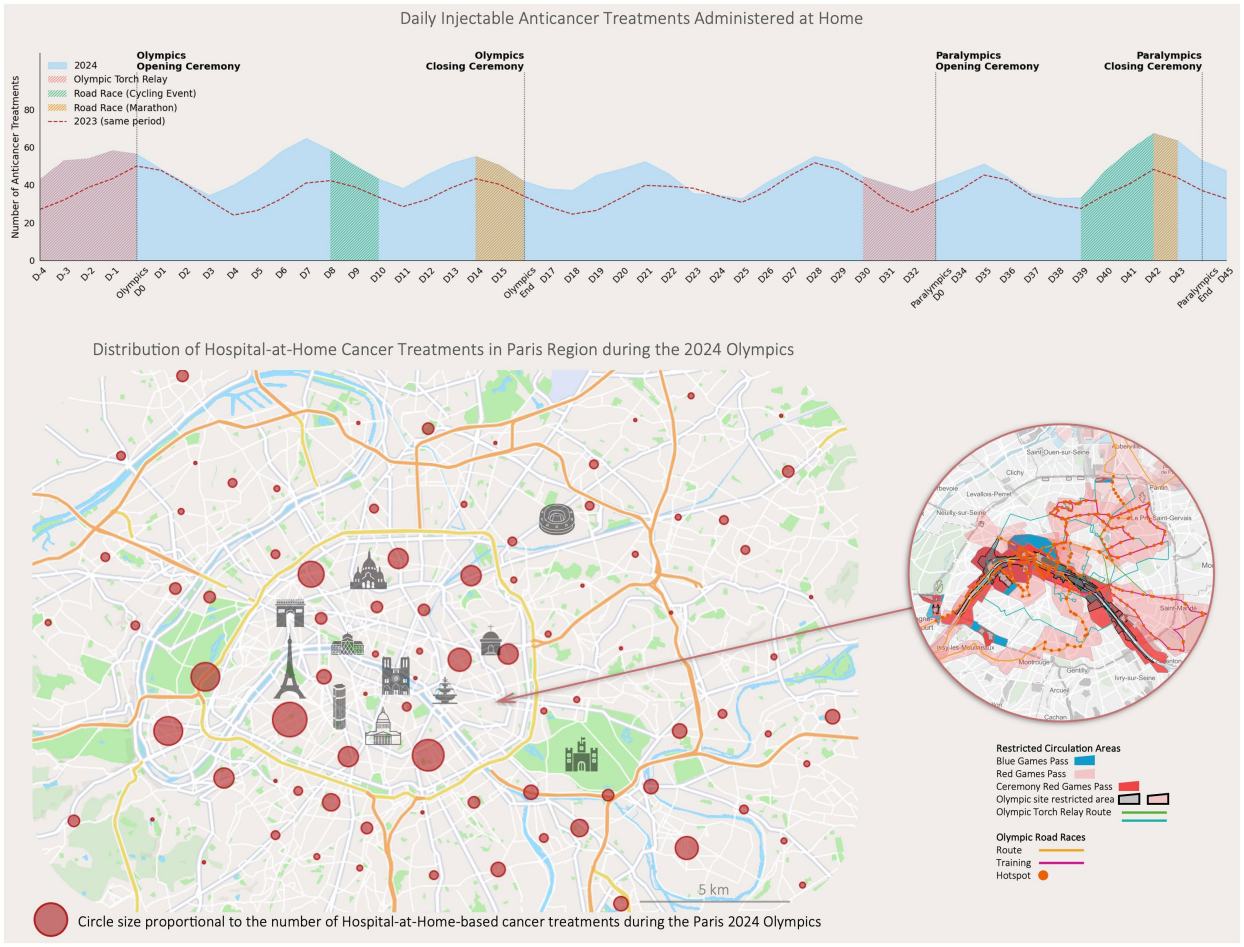
Daily injectable anticancer treatments administered at home and distribution of treatments in the paris region during the 2024 Olympics. The upper graph illustrates the number of injectable anticancer treatments administered at home daily during the 2024 Paris Olympics, with specific Olympic events. Data from the same period in 2023 are included. The lower map shows the geographic distribution of Hospital at Home cancer treatments across the Paris region during the Olympics. Circle sizes are proportional to the number of treatments administered in each area. Restricted circulation zones and Olympic-related routes are overlaid, highlighting areas affected by increased traffic restrictions. The inset focuses on high-traffic zones in Paris.

From July 26 to September 8, 2024, a total of 535 patients received cancer treatments through the HaH program (Figure 1). The age range was from 3 to 94 years, with a median age of 72 years (IQR 60–79), including 29 pediatric cases (Figure 2A). They represented 4.76% of the total cancer patients treated across Greater Paris University Hospitals for chemotherapy/immunotherapy sessions, which marked a significant increase compared to the same period in 2023 (455 patients, or 3.9%, *p* < 0.05).

During this period, a total of 1,946 chemotherapy/immunotherapy sessions were administered in HaH (Figure 2B). The primary indications were multiple myeloma (*n* = 235), myeloid neoplasms (*n* = 175), gynecologic cancers (including breast, ovarian, uterine, and endometrial cancers, *n* = 56), lymphoma (*n* = 45), and other cancers (lung, liver, stomach, testicular, skin, sarcoma, *n* = 24).

The treatments involved 31 different drugs, with the most frequently being azacitidine (*n* = 1,025), daratumumab (*n* = 248), bortezomib (*n* = 224), cytarabine (*n* = 65), arsenic (*n* = 63), pertuzumab trastuzumab (*n* = 46), carfilzomib (*n* = 42), teclistamab (*n* = 34), and blinatumomab (*n* = 33).

By comparing scheduled versus actual administration times, healthcare providers systematically documented any deviations. No significant delays or patient harm were reported within the HaH program throughout the disruption period caused by the OG & PG.

## Discussion

AP-HP’s HaH services successfully implemented a strategy to ensure the continuity of cancer treatments during the Paris 2024 Games. Key obstacles included maintaining timely communication amid rapidly changing traffic conditions and managing drug delivery logistics under tight deadlines. HaH addressed these challenges with adaptive measures supported by a dedicated management team operating daily, ensuring effective coordination among healthcare personnel.

The success of this initiative highlights the critical importance of flexibility, real-time problem-solving, and patient-centered planning when facing unprecedented logistical challenges. Although these measures were developed specifically for the unique circumstances of the OG, they offer valuable lessons for routine healthcare delivery (5, 6). Many of the strategies employed, including real-time adaptation and optimized care delivery schedules, can be integrated into standard HaH operations (7, 8). By incorporating these practices, healthcare systems can enhance efficiency, resilience, and patient satisfaction, even in non-emergency settings. This experience offers valuable insights for other cities managing large-scale events or seeking to improve healthcare delivery.

## Data Availability

The raw data supporting the conclusions of this article will be made available by the authors, without undue reservation.
